# Diurnal and Daily Symptom Variation in Patients with End Stage Kidney Disease: An Ecological Momentary Assessment Study

**DOI:** 10.2215/CJN.0000000000000524

**Published:** 2024-07-16

**Authors:** Cramer J. Kallem, Alaa A. Alghwiri, Jonathan Yabes, Sarah Erickson, Zhuoheng Han, Maria-Eleni Roumelioti, Jennifer L. Steel, Manisha Jhamb, Mark Unruh

**Affiliations:** 1 Renal-Electrolyte Division, Department of Medicine, University of Pittsburgh School of Medicine, Pittsburgh, Pennsylvania; 2 Division of General Internal Medicine, Center for Research on Heath Care Data Center, Department of Medicine and Biostatistics, University of Pittsburgh, Pittsburgh, Pennsylvania; 3 Department of Psychology, University of New Mexico, University of New Mexico, Albuquerque, New Mexico; 4 Division of Nephrology, Department of Internal Medicine, University of New Mexico School of Medicine, Albuquerque, New Mexico; 5 Department of Surgery, University of Pittsburgh School of Medicine, Pittsburgh, Pennsylvania; 6 Department of Psychiatry, University of Pittsburgh School of Medicine, Pittsburgh, Pennsylvania; 7 Department of Psychology, University of Pittsburgh School of Medicine, Pittsburgh, Pennsylvania

**Keywords:** clinical nephrology, cognition, depression, dialysis, ESKD, hemodialysis, patient self-assessment, quality of life

## Abstract

**Key Points:**

- Patients treated with maintenance hemodialysis report a higher symptom burden on the days they receive hemodialysis treatment, compared with non-hemodialysis days.
- The severity of physical, cognitive, and mood symptoms can vary diurnally, and the pattern of this variation may differ between hemodialysis and non-hemodialysis days.
- Ecological momentary assessment can provide insights into the complex, dynamic symptom experiences of patients on hemodialysis.

**Background:**

Patients with ESKD on hemodialysis experience a high symptom burden, which is compounded by unpredictable fluctuations in symptom severity. Few studies have used ecological momentary assessment to determine how symptoms vary over time. This study aimed to characterize the diurnal and day-to-day variability in symptoms among patients receiving hemodialysis.

**Methods:**

Patients enrolled in the Technology-Assisted Collaborative Care trial rated the intensity of physical, cognitive, and mood symptoms using an automated telephone-administered version of the Daytime Insomnia Symptom Scale at four time points (morning, early afternoon, late afternoon, evening) for seven consecutive days at baseline. Confirmatory factor analysis was used to verify the original four-factor solution for the Daytime Insomnia Symptom Scale: sleepiness/fatigue (SF), alert cognition (AC), positive mood (PM), and negative mood (NM). Symptom domain scores were calculated for each time point, and mixed modeling with random patient effects was used to examine differences in daily symptoms at daily time points between hemodialysis and non-hemodialysis days after controlling for age, sex, race, and comorbidity burden.

**Results:**

One hundred sixty patients were enrolled (mean±SD age 58±14 years, 45% women, 52% White). Diurnal symptom variation existed; trends were nonlinear and differed by hemodialysis versus non-hemodialysis days. Day-to-day symptom variation also existed; patients endorsed better physical, cognitive, and mood states (*i.e*., higher AC and PM) as well as lower symptom burden (*i.e*., lower SF and NM) on non-hemodialysis days compared with hemodialysis days at all time points. The greatest day-to-day mean differences (MDs) were observed in the early afternoon for all symptom domains: AC (MD=0.17 *P* < 0.001), PM (MD=0.28, *P* < 0.001), SF (MD=−0.66, *P* < 0.001), and NM (MD=−0.26, *P* < 0.001).

**Conclusions:**

Patients with ESKD demonstrate diurnal variation in symptoms and greater symptom burden on hemodialysis days compared with non-hemodialysis days, with the most extreme differences in symptom severity occurring in the early afternoon.

**Clinical Trial registration number:**

ClinicalTrials.gov NCT03440853.

## Visual Abstract

**Figure:**
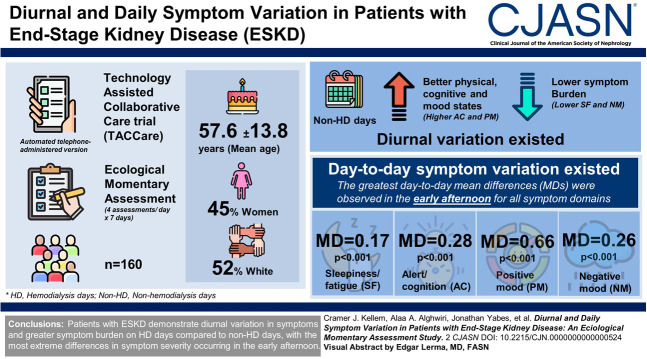

## Introduction

Patients with ESKD receiving maintenance hemodialysis treatment often carry a high symptom burden, including both physical (*e.g*., fatigue, poor sleep) and mood (*e.g*., depression, anxiety) symptoms.^1–3^ In fact, most patients with ESKD experience comorbid symptoms, which negatively affect their quality of life.^2,4,5^ Although patients frequently cite improving symptom management as being among their top priorities,^1,2,6^ adverse symptoms of ESKD and side effects from treatment remain frequently under-recognized and undertreated by providers.^5,7^ Identifying effective symptom management strategies may be particularly challenging for patients on hemodialysis because they demonstrate wide interpersonal variability in symptom burden and recovery from dialysis.^8^

Although patients with ESKD are known to have complex, burdensome, and persistent symptom experiences, very few studies have examined how symptoms may vary diurnally (*i.e*., throughout the day) or from day to day (*i.e*., on dialysis versus non-dialysis days) in these patients.^9^ This gap is notable because patients with ESKD frequently experience fluctuating symptoms^10^ and symptom unpredictability (*i.e*., unpredictable timing of onset and duration), which seem to be major contributors to symptom distress.^2^

Ecological momentary assessment (EMA), which involves repeated sampling of participants' symptoms in real time, has the potential to provide valuable insights into the dynamics of ESKD symptoms.^11^ In addition to providing data at multiple time points, EMA helps to overcome challenges related to long-term recall bias, which is common in all populations,^11^ but may be further warranted secondary to the cognitive impairments associated with ESKD.^12,13^ In fact, findings from a small study by Brys *et al.*^14^ suggest patients may tend to overestimate symptom severity when assessed using retrospective self-report measures compared with EMA.^14^ Increased understanding of the temporal factors associated with symptom burden has the potential to improve provider assessment of symptoms and increase the efficacy of symptom management interventions.

In our small prior study,^15^ we observed significant diurnal and day-to-day symptom variability on a number of Daytime Insomnia Symptom Scale (DISS)^16^ items and on a fatigue–sleepiness–exhaustion composite score; however, this was the only symptom domain examined.^15^ Only two other studies have examined symptoms of ESKD and side effects of hemodialysis using EMA.^14,15,17^ Both these studies were limited by small sample sizes and limited analysis of day-to-day differences or diurnal patterns in symptoms.^17^ Additional studies are needed to further characterize the variability of the multitude of symptoms and side effects in dialysis-dependent ESKD.

To address the gap in knowledge regarding the experience of patients undergoing thrice-weekly dialysis, this study aimed to (*1*) investigate the correlation of patient-reported symptoms measured using EMA with validated retrospective self-report measures and (*2*) describe day-to-day and diurnal variability in physical, cognitive and mood symptoms in a large well-characterized diverse sample of patients with ESKD.

## Methods

### Setting and Participants

This study is a secondary analysis of data collected in the Technology-Assisted Stepped Collaborative Care (TĀCcare) trial.^18,19^ Participants were recruited in the United States from dialysis clinics in the states of Pennsylvania and New Mexico between March 2019 and December 2021. All patients were adults with ESKD on maintenance hemodialysis who screened positive for depression, pain, or fatigue. The study was approved by the institutional review boards at the University of New Mexico and the University of Pittsburgh. TĀCcare trial participants provided written informed consent before the administration of questionnaires; a detailed description of the study procedures has been published elsewhere.^18,19^ For the current study, we used the data collected at baseline, before randomization in the TĀCcare trial.^18^

### Data Collection

Sociodemographic and clinical data were collected from computer-assisted patient interviews and medical records. Demographic variables included age, sex, race, and ethnicity. These data were collected to allow for the examination of differences in outcomes on the basis of sociodemographic and clinical characteristics and to allow for the inclusion of these factors in statistical models. Patients received four daily EMA automated telephone calls, which occurred at random times within three-hour windows in the morning (9:00 am to 11:59 am), early afternoon (12:00 pm to 2:59 pm), late afternoon (3:00 pm to 5:59 pm), and evening (6:00 pm to 8:59 pm) for seven consecutive days. When patients were unable to respond to calls (because of hospitalization or technical issues), additional days were added.

### Measures

#### DISS

The DISS is a 19-item self-report measure of physical, cognitive, and mood symptoms that was adapted to be administered by automated telephone calls.^16^ Patients responded to short questions (*e.g*., “How anxious do you feel right now?”) using a seven-point Likert scale, with higher scores indicating higher levels of the symptom. Item-level scores can range from one to seven. In patients with primary insomnia and in the general population, four principal components have been identified from the DISS: alert cognition (AC), sleepiness/fatigue (SF), positive mood (PM), and negative mood (NM).^16^ In our prior study of patients with ESKD that focused on the SF factor, EMA using DISS was successful in demonstrating its day-to-day and diurnal variation.^1^

#### Retrospective Self-Report Measures

The following questionnaires were administered at baseline by trained interviewers who used computer-assisted telephone interviewing: Functional Assessment of Chronic Illness Therapy—Fatigue Scale, a 13-item self-report measure of fatigue and its effect on daily functioning within the past week (score range=0–52) with lower scores indicating greater fatigue^20^; Beck Depression Inventory II, a 21-item self-report measure of depressive symptoms within the past 2 weeks (score range=0–63) with higher scores indicating greater depressive symptoms^21^; Generalized Anxiety Disorder 7 (GAD-7), a seven-item self-report measure of anxiety symptoms within the past week (score range=0–21) with higher scores indicating greater anxiety^22^; and Pittsburgh Sleep Quality Index (PSQI), a 19-item self-report measure of sleep within the past month, which includes seven components (sleep quality, sleep latency, sleep duration, sleep efficiency, sleep disturbance, sleep medication use, daytime dysfunction) and yields a total composite score (score range=0–21) with higher scores indicating poorer sleep.^23^

### Statistical Analyses

Descriptive statistics were used to examine sample characteristics. For DISS, symptom domain scores were calculated for all patients at all time points. Symptom domain scores ranged from one to seven and were derived by calculating the mean of all items within that factor. Confirmatory factor analysis (CFA) was performed to test whether the four-factor solution for the DISS adequately fit our data with patients with ESKD on hemodialysis. We did not include the weary item in the CFA because it was not included in the original four-factor solution (because of factor weight <0.4 on all factors).^16^

Subgroup analyses were performed to evaluate differences in symptom domain scores by age, sex, race, ethnicity, Charlson Comorbidity Index (CCI) score (a measure used to quantify burden related to comorbid medical conditions),^24^ dialysis vintage, dialysis shift (morning versus mid-day), and dialysis schedule (Monday-Wednesday-Friday versus Tuesday-Thursday-Saturday). Pearson correlation coefficients were computed to examine correlations between DISS symptom domain scores and baseline questionnaire scores. Mixed modeling with random patient effects was used to examine diurnal (i.e., time point to time point) and day to day (i.e., HD versus non-HD days) variation in symptoms after controlling for age, gender, race, and CCI score. All analyses were performed using R version 4.3.3 (R Foundation for Statistical Computing).

## Results

### Sample Characteristics

A total of 160 patients were enrolled in the study (mean age=58; SD=14). Most of the patients were male (*n*=88; 55%) and White (*n*=83; 52%). Table 1 summarizes the sample characteristics. Among the 160 patients, EMA data call completion rate was 82%, with 3664 of the 4480 total possible calls completed. The average number of calls completed per patient was 23 (of max 28 possible calls). Most of the patients completed EMA calls using a mobile telephone (*n*=138, 86.3%) instead of a landline telephone (*n*=22, 14%). The average EMA telephone call times were as follows: morning (mean call time=10:28 am±49 minutes), early afternoon (mean call time=1:20 pm±64 minutes), late afternoon (mean call time=4:24 pm±49 minutes), and evening (mean call time=7:08 pm±4 minutes).

**Table 1:** Sample characteristics

Characteristic	Sample No.=160 (Mean [SD] or No. [%])
Age	57.6 (14)
**Sex**	
Female	72 (45)
Male	88 (55)
**Race**	
American Indian	21 (13)
Black	45 (28)
White	83 (52)
Other (>1 race/unknown)	11 (7)
**Ethnicity**	
Hispanic	28 (18)
Non-Hispanic	125 (78)
Missing	7 (4)
CCI	4.7 (2)
Dialysis vintage (yr)	4.1 (4)
FACIT-F	27.0 (11)
BDI-II	21.0 (11)
GAD-7	6.0 (5)
PSQI	9.0 (3)
No. of EMA calls completed per person (maximum possible calls=28)	22.9 (5)
% of EMA calls completed per person	82
Completed ≥50% of EMA calls	152 (95)

Functional Assessment of Chronic Illness Therapy—Fatigue scores range from 0 to 52 with lower scores indicating greater fatigue; Beck Depression Inventory II scores range from 0 to 63 with higher scores indicating greater depressive symptoms; Generalized Anxiety Disorder-7 scores range from 0 to 21 with higher scores indicating greater anxiety; Pittsburgh Sleep Quality Index scores range from 0 to 21 with higher scores indicating poorer sleep. CCI, Charlson Comorbidity Index; EMA, ecological momentary assessment; FACIT-F, Functional Assessment of Chronic Illness Therapy—Fatigue; BDI-II, Beck Depression Inventory II; GAD-7, Generalized Anxiety Disorder-7; PSQI, Pittsburgh Sleep Quality Index.

### CFA of DISS

CFA revealed the four-factor solution for the DISS previously described in patients with primary insomnia and healthy controls^16^ (AC, SF, PM, and NM) adequately fit our data (standardized root mean square residual=0.06; Tucker Lewis Index=0.91; see Table 2 for factor loadings).

**Table 2:** Confirmatory factor analysis: unstandardized factor loadings for Daytime Insomnia Symptom Scale items

Symptom Domain Items	Factor Loading	Standard Error	Z-value	*P* Value
**Alert Cognition**				
Forgetful	−0.80	0.03	−26.96	<0.001
Clear-headed	1.23	0.03	43.66	<0.001
Concentrate	1.13	0.03	43.54	<0.001
Effort	−0.81	0.03	−24.42	<0.001
Alert	1.14	0.03	38.21	<0.001
**Sleepiness/Fatigue**				
Fatigued	1.42	0.03	48.26	<0.001
Sleepy	1.54	0.03	50.50	<0.001
Exhausted	1.75	0.03	61.11	<0.001
**Positive Mood**				
Relaxed	1.23	0.03	41.51	<0.001
Energetic	1.10	0.03	38.88	<0.001
Calm	1.25	0.03	44.47	<0.001
Happy	1.28	0.03	47.35	<0.001
Efficient	1.16	0.03	43.55	<0.001
**Negative Mood**				
Anxious	1.16	0.03	44.31	<0.001
Stressed	1.55	0.03	61.10	<0.001
Tense	1.52	0.02	63.50	<0.001
Sad	1.01	0.03	38.72	<0.001
Irritable	1.42	0.03	53.40	<0.001

### Correlation between DISS Domains and Self-Report Measures

There were significant correlations between DISS symptom domain scores and retrospective self-report measures completed at baseline, and strength of correlation was moderate for most (Table 3). Both AC and PM were significantly correlated with lower self-reported fatigue, anxiety, and depressive symptoms and with greater overall sleep quality. Both SF and NM were significantly correlated with greater fatigue, anxiety, and depressive symptoms and with lower overall sleep quality.

**Table 3:** Unadjusted Pearson correlations between average Daytime Insomnia Symptom Scale symptom domain scores and retrospective self-report measures

Measures	Pearson's *r*	*P* Value	Effect Size Interpretation
**Alert Cognition**			
FACIT-F	0.31	<0.001	Moderate
BDI-II	−0.32	<0.001	Moderate
GAD-7	−0.34	<0.001	Moderate
PSQI	−0.21	<0.001	Weak
**Sleepiness/Fatigue**			
FACIT-F	−0.50	<0.001	Moderate
BDI-II	0.40	<0.001	Moderate
GAD-7	0.38	<0.001	Moderate
PSQI	0.31	<0.001	Moderate
**Positive Mood**			
FACIT-F	0.54	<0.001	Strong
BDI-II	−0.52	<0.001	Strong
GAD-7	−0.42	<0.001	Moderate
PSQI	−0.34	<0.001	Moderate
**Negative Mood**			
FACIT-F	−0.39	<0.001	Moderate
BDI-II	0.56	<0.001	Strong
GAD-7	0.63	<0.001	Strong
PSQI	0.34	<0.001	Moderate

Lower scores on Functional Assessment of Chronic Illness Therapy—Fatigue indicate greater fatigue. Higher scores on Beck Depression Inventory II, General Anxiety Disorder 7, and Pittsburgh Sleep Quality Index indicate higher depression, anxiety, and poorer sleep quality, respectively. FACIT-F, Functional Assessment of Chronic Illness Therapy—Fatigue; BDI-II, Beck Depression Inventory II; GAD-7, Generalized Anxiety Disorder-7; PSQI, Pittsburgh Sleep Quality Index.

### Symptom Domain Scores

**Figure 1:**
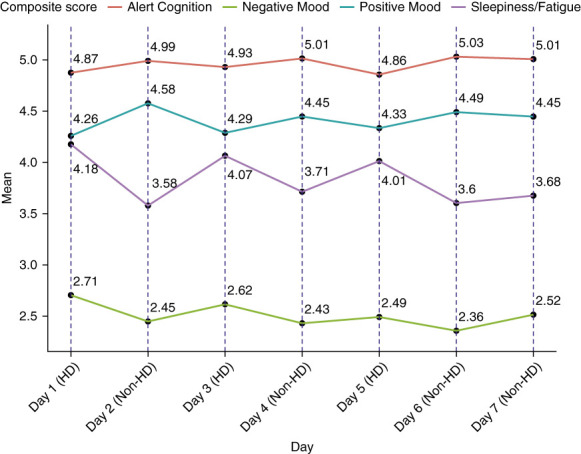
**Mean symptom domain scores by day of the week.** For patients on a Monday-Wednesday-Friday dialysis schedule, day 1=Monday; for patients on a Tuesday-Thursday-Saturday dialysis schedule, day 1=Tuesday. For AC and PM, higher score is better; for NM and SF, higher score is worse. AC, alert cognition; NM, negative mood; PM, positive mood; SF, sleepiness/fatigue.

After confirming the four-factor solution, symptom domain scores were computed. The mean symptom domain scores on hemodialysis and non-hemodialysis days, respectively, were as follows: AC (4.9 [SD=0.7], 5.0 [SD=0.8]), SF (4.1 [SD=1.2], 3.7 [SD=1.2]), PM (4.3 [SD=1.0], 4.5 [SD=1.0]), and NM (2.6 [SD=1.2], 2.5 [SD=1.1]). See Figure 1 for average symptom domain scores by day of the week.

In subgroup analyses (Supplemental Figure 1, A–D), average PM scores were significantly higher among the other race category (American Indian, >1 race, or unknown) (mean=4.89±1.14) as compared with Black (mean=4.36±0.93) or White (mean=4.30±0.91, *P* = 0.03) patients. In addition, average NM scores were significantly higher for patients younger than 65 years (mean=2.64±1.17) than older patients (mean=2.21±0.95, *P* = 0.04). There were no other significant differences in other domain scores by age, sex, race, ethnicity, CCI, or dialysis vintage. There were no significant differences in symptom domain scores between patients on a Monday-Wednesday-Friday hemodialysis schedule (*N*=103) and those on a Tuesday-Thursday-Friday hemodialysis schedule (*n*=57; *P* value range = 0.30 to >0.99). There were also no significant differences in symptom domain scores between patients who receive hemodialysis in the early morning (*N*=75) from those who receive hemodialysis in the afternoon (*n*=80; *P* value range = 0.06–0.70); due to the limited number of patients who receive hemodialysis in the evening (*n*=5), they were excluded from this between-group comparison.

### Diurnal Patterns in Symptoms

After adjusting for age, race, sex, and CCI, diurnal patterns in symptom domain scores were observed. On hemodialysis days, there were significant changes in symptoms between daily time points for SF (increase from morning to early afternoon [mean difference (MD)=0.41, 95% confidence interval (CI) 0.23 to 0.60, *P* < 0.001], increase from late afternoon to evening [MD=0.22, 95% CI (0.04 to 0.39), *P*=0.02]) and NM (increase from morning to early afternoon [MD=0.17, 95% CI (0.03 to 0.30), *P* = 0.02], decrease from late afternoon to evening [MD=−0.16, 95% CI (−0.26 to −0.01), *P* = 0.04]). Of note, the average time of hemodialysis for patients overlapped with the morning and early afternoon periods. On non-hemodialysis days, there was a significant change in SF (increase from late afternoon to evening [MD=0.35, 95% CI (0.20 to 0.50), *P* < 0.001]). For all between-time point changes in symptom domain scores, see Supplemental Tables 1 and 2 for unadjusted and adjusted estimates, respectively.

### Comparison of Average Symptom Severity on Dialysis versus Non-Dialysis Days

**Figure 2:**
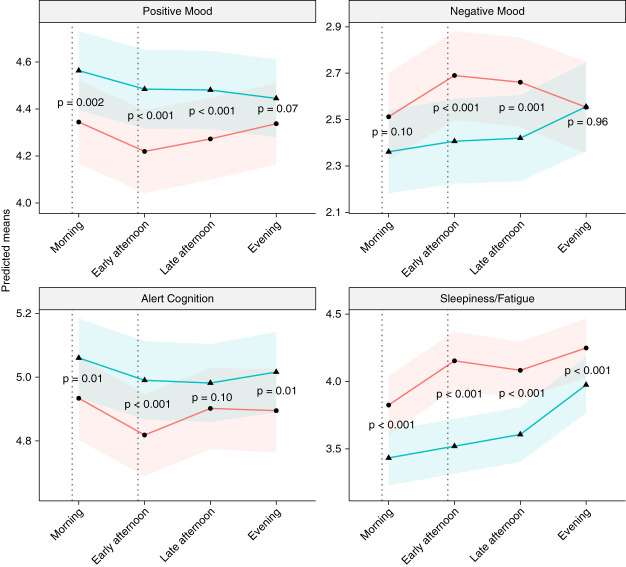
**Predicted means of symptom domain scores by time of day on dialysis versus non-dialysis days after controlling for age, race, sex, and comorbidity burden.** *P* values are based on adjusted mixed model comparisons of mean symptom domain scores at each time point on dialysis versus non-dialysis days. Dotted vertical lines indicate the average start and end times for hemodialysis treatments (mean dialysis start time=8:52 am±140 minutes, mean dialysis end time=12:56 pm±141 minutes). For AC and PM, higher score is better; for NM and SF, higher score is worse.

After adjusting for age, race, sex, and CCI, on hemodialysis days, patients had higher SF and NM scores and lower AC and PM scores at all time points compared with non-hemodialysis days. These between-day differences were statistically significant for the following domains and time points: AC (morning [MD=0.13, 95% CI (0.04 to 0.22), *P* = 0.01], early afternoon [MD=0.17, 95% CI (0.09 to 0.26), *P* < 0.001], evening [MD=0.11, 95% CI (0.02 to 0.19), *P* = 0.01]), SF (morning [MD=−0.33, 95% CI (−0.50 to −0.16), *P* < 0.001], early afternoon [MD=−0.65, 95% CI (−0.81 to −0.50), *P* < 0.001], late afternoon [MD=−0.44, 95% CI (−0.60 to −0.28), *P* < 0.001], evening [MD=−0.31, 95% CI (−0.47 to −0.16), *P* < 0.001]), PM (morning [MD=2.00, 95% CI (0.07, 0.33), *P*=0.002], early afternoon [MD=0.28, 95% CI (0.16, 0.40), *P*<0.001], late afternoon [MD=0.22, 95% CI (0.10, 0.34), *P*<0.001]), and NM (early afternoon [MD=−0.26, 95% CI (−0.38 to −0.13), *P* < 0.001], late afternoon [MD=−0.21, 95% CI (−0.32 to −0.09), *P* = 0.001]). The greatest between-day MD occurred in the early afternoon for all symptom domains. See Figure 2 and Supplemental Table 4 for adjusted comparisons of symptom domain scores at all time points on hemodialysis and non-hemodialysis days; see Supplemental Table 3 for unadjusted comparisons. See Supplemental Figures 2 and 3 for symptom domain scores at all time points for patients on an early morning hemodialysis schedule and patients on an afternoon hemodialysis schedule separately.

## Discussion

This was the largest study to date to characterize symptom variability using EMA in a diverse sample of patients with ESKD. We observed significant diurnal and hemodialysis day versus non-hemodialysis day variation in symptom burden. Our findings are consistent with the complex, dynamic symptom experiences described in previous qualitative studies, wherein patients report multiple concurrent symptoms that tend to wax and wane over time.^2,9^

Patients demonstrated greater diurnal symptom variation on hemodialysis days, with a significant increase in both SF and NM from morning to early afternoon, which coincided with the average hemodialysis time. In addition, they had a significant increase in SF from late afternoon to evening. These findings align with those of previous studies demonstrating that fatigue and depressive symptoms tend to cluster together in patients with kidney disease and other chronic medical conditions and may be related to hemodialysis treatment itself.^25–28^ By contrast, on non-hemodialysis days, a late afternoon to evening increase in SF was the only statistically significant change in DISS symptom domain scores.

Of note, diurnal patterns in patients' symptom burden differed between hemodialysis and non-hemodialysis days. This was most evident for the NM symptom domain score. On hemodialysis days, patients' NM symptoms increased from morning to early afternoon, remained elevated through the late afternoon, and then significantly improved in the evening. By contrast, on non-hemodialysis days, NM symptoms tended to be less intense, remained relatively stable throughout the day, and then worsened in the evening. An inverse, yet less extreme, pattern in the PM symptom domain score was also observed.

Although we did observe an increase in SF scores from late afternoon to evening on both hemodialysis and non-hemodialysis days, this increase was greater on hemodialysis days. Furthermore, patients also reported increases in cognitive symptoms (*e.g*., forgetfulness, poor concentration, reduced alertness) from late afternoon to evening on hemodialysis days. Of note, patients endorsed the greatest spike in symptom burden in the early afternoon on hemodialysis days, which corresponds with either the intradialytic period or within the first few hours of the postdialysis period for most patients. These findings are consistent with the washed-out feeling many patients describe on hemodialysis days^29^ and with the conclusions made at the postdialysis fatigue National Institutes of Health conference advocating for use of the term postdialysis syndrome because the patients may experience worsening in a range of symptoms after hemodialysis.^30^ Further research is needed on understanding patient symptoms, mechanistic underpinnings, and possible intervention strategies to improve patient experience in the postdialysis period. Future studies should use EMA to examine whether symptom variation may differ by dialysis treatment modality (*e.g*., peritoneal dialysis, self-care hemodialysis, home hemodialysis, nocturnal hemodialysis).

Overall, our findings highlight the utility of EMA to gain valuable insights into the symptom experiences of patients with ESKD. The high completion rate of EMA calls (82%) provides support for the feasibility and acceptability of this approach to data collection. Our results also provide support for the validity of the DISS symptom domain scores as a measure of self-reported symptoms in patients receiving hemodialysis because we found significant correlations between DISS symptom domain scores and validated retrospective self-report measures of closely related constructs (*i.e*., higher levels of depression [Beck Depression Inventory II] and anxiety [GAD-7] were associated with greater NM and lower PM; higher levels of fatigue [Functional Assessment of Chronic Illness Therapy—Fatigue] and poor sleep [PSQI] were associated with greater SF and lower AC).

In addition, our findings have important clinical implications which could help inform the development or refinement of symptom management interventions. For example, energy conservation and physical activity interventions are among the most empirically supported treatment approaches for fatigue in patients with chronic medical conditions.^31^ Considering the trends in SF severity in our sample and the practical constraints associated with lengthy dialysis treatments, future studies should examine the efficacy of interventions that aim to increase the use of energy conservation strategies on hemodialysis days and increase physical activity on non-hemodialysis days in patients with ESKD. In addition, we recently demonstrated that a collaborative care intervention, based on cognitive-behavioral therapy principles, delivered during hemodialysis can meaningfully reduce fatigue and pain.^18^ Future studies on interventions that specifically target acute symptom exacerbations like those observed in early afternoon on hemodialysis days are needed and should use EMA to examine the effectiveness of such interventions.

While this work demonstrated significant diurnal and day-to-day variations in a broad range of physical, cognitive, and mood symptoms in patients with ESKD on hemodialysis, there are limitations that should be noted. All patients in our study screened positive for at least one of the three symptoms—depression, pain or fatigue at enrollment—because this was a requirement for inclusion in the TĀCcare trial. Thus, our findings may not be generalizable to patients without these symptoms. However, given the high prevalence of these symptoms in patients on hemodialysis^2,3,32–34^ and a screen positive rate of 76% for at least one symptom in the TĀCcare trial,^35^ our findings are highly likely to be relevant to most patients on hemodialysis. In addition, some of our data were collected during the coronavirus disease 2019 pandemic, and it is unclear whether, or to what extent, coronavirus disease 2019 infections or vaccinations could have affected patient-reported symptoms in this study. Interpretation of our findings is limited to four daily time points (morning, early afternoon, late afternoon, evening). Including additional time points (*e.g*., every 2 or 3 hours during waking hours) would provide a clearer picture of symptom variation patterns and allow for a closer examination of the syndrome these patients experience during the postdialysis period. However, the participant burden may be such that this would not yield additional information (*i.e*., because of a lower response rate). Future studies should examine whether collecting EMA data using mobile phone applications could reduce participant burden and allow for more frequent assessment of symptoms; such approaches would also allow for different response formats (*e.g*., visual analog scale), which could increase accessibility. Another limitation is the inclusion of only a subset of ESKD symptoms. Future EMA studies may include items that assess additional symptoms, especially body aches, itching, and cramping, because these have also been cited as being among the most bothersome symptoms by patients with ESKD, in part, because of the unpredictability of their occurrence.^2^ Despite these limitations, this study has a number of strengths including a large and diverse sample of patients with ESKD as well as the use of validated EMA and retrospective self-report symptom measures with a high data completion rate.

Additional research using EMA data collection methods to examine diurnal and day-to-day patterns of symptom variation has the potential to improve symptom assessment and management for patients with ESKD in a number of ways. Gathering detailed information on the frequency, intensity, duration, and timing of symptoms could be used to identify subgroups of patients with similar symptom patterns, allow for between-subgroup comparisons of treatment response, and potentially inform best practices for treatment selection depending on patients' symptom patterns. EMA also has the potential to inform a number of innovative precision medicine approaches, beyond treatment selection, including personalized recommendations for patients (*e.g*., optimal timing of behavioral self-management skills or medications) and the systematic adjustment of the treatment approach on the basis of an ongoing, real-time assessment of treatment response.

## Supplementary Material

## Data Availability

Anonymized data created for the study are or will be available in a persistent repository upon publication. Analyzable Data. Statistical Analysis Plan. GitHub. The data will be reposited in Github after final publication.

## Supplemental Material

Supplemental Table 1. Unadjusted changes in symptom domain scores between time points.

Supplemental Table 2. Changes in symptom domain scores between time points after adjusting for age, race, sex, and Charlson Comorbidity Index.

Supplemental Table 3. Unadjusted mean difference in symptom domain scores on dialysis versus non-dialysis days at all time points.

Supplemental Table 4. Mean difference in symptom domain scores on dialysis versus non-dialysis days at all time points after adjusting for age, race, sex, and Charlson Comorbidity Index.

Supplemental Figure 1A. Subgroup analysis of DISS alert cognition scores.

Supplemental Figure 1B. Subgroup analysis of DISS sleepiness/fatigue scores.

Supplemental Figure 1C. Subgroup analysis of DISS positive mood scores.

Supplemental Figure 1D. Subgroup Analysis of DISS negative mood scores.

Supplemental Figure 2. Predicted means of symptom domain scores for patients on early morning hemodialysis by time of day on dialysis versus non-dialysis days after controlling for age, race, sex, and comorbidity burden.

Supplemental Figure 3. Predicted means of symptom domain scores for patients on afternoon hemodialysis by time of day on dialysis versus non-dialysis days after controlling for age, race, sex, and comorbidity burden.

## References

[1] Mehrotra R Davison SN Farrington K, . Managing the symptom burden associated with maintenance dialysis: conclusions from a kidney disease: improving global outcomes (KDIGO) controversies conference. Kidney Int. 2023;104(3):441–454. doi:10.1016/j.kint.2023.05.019 37290600

[2] Flythe JE Hilliard T Castillo G, . Symptom prioritization among adults receiving in-center hemodialysis: a mixed methods study. Clin J Am Soc Nephrol. 2018;13(5):735–745. doi:10.2215/CJN.10850917 29559445 PMC5969481

[3] Fletcher BR Damery S Aiyegbusi OL, . Symptom burden and health-related quality of life in chronic kidney disease: a global systematic review and meta-analysis. PLoS Med. 2022;19(4):e1003954. doi:10.1371/journal.pmed.1003954 35385471 PMC8985967

[4] Murtagh FEM Addington-Hall J Higginson IJ. The prevalence of symptoms in end-stage renal disease: a systematic review. Adv Chronic Kidney Dis. 2007;14(1):82–99. doi:10.1053/j.ackd.2006.10.001 17200048

[5] Li H Xie L Yang J Pang X. Symptom burden amongst patients suffering from end-stage renal disease and receiving dialysis: a literature review. Int J Nurs Sci. 2018;5(4):427–431. doi:10.1016/j.ijnss.2018.09.010 31406859 PMC6626284

[6] Manns B Hemmelgarn B Lillie E, . Setting research priorities for patients on or nearing dialysis. Clin J Am Soc Nephrol. 2014;9(10):1813–1821. doi:10.2215/CJN.01610214 24832095 PMC4186509

[7] Claxton RN Blackhall L Weisbord SD Holley JL. Undertreatment of symptoms in patients on maintenance hemodialysis. J Pain Symptom Manage. 2010;39(2):211–218. doi:10.1016/j.jpainsymman.2009.07.003 19963337

[8] Caplin B Kumar S Davenport A. Patients’ perspective of haemodialysis-associated symptoms. Nephrol Dial Transplant. 2011;26(8):2656–2663. doi:10.1093/ndt/gfq763 21212166

[9] Ng MSN Wong CL Ho EHS Hui YH Miaskowski C So WKW. Burden of living with multiple concurrent symptoms in patients with end-stage renal disease. J Clin Nurs. 2020;29(13-14):2589–2601. doi:10.1111/jocn.15282 32279368

[10] Parfrey PS Vavasour HM Henry S Bullock M Gault MH. Clinical features and severity of nonspecific symptoms in dialysis patients. Nephron. 1988;50(2):121–128. doi:10.1159/000185141 3065660

[11] Shiffman S Stone AA Hufford MR. Ecological momentary assessment. Annu Rev Clin Psychol. 2008;4:1–32. doi:10.1146/annurev.clinpsy.3.022806.091415 18509902

[12] Tamura MK Yaffe K. Dementia and cognitive impairment in ESRD: diagnostic and therapeutic strategies. Kidney Int. 2011;79(1):14–22. doi:10.1038/ki.2010.336 20861818 PMC3107192

[13] Henry SL Jamner LD Choi SE Pahl MV. The effect of the interdialytic interval on cognitive function in patients on haemodialysis. J Ren Care. 2018;44(1):44–51. doi:10.1111/jorc.12231 29271080 PMC6065249

[14] Brys ADH Stifft F Van Heugten CM Bossola M Gambaro G Lenaert B. Unraveling fatigue in hemodialysis patients: comparing retrospective reports to real-time assessments with an mHealth experienced sampling method. J Pain Symptom Manage. 2020;60(6):1100–1108.e2. doi:10.1016/j.jpainsymman.2020.06.042 32645453

[15] Abdel-Kader K Jhamb M Mandich LA, . Ecological momentary assessment of fatigue, sleepiness, and exhaustion in ESKD. BMC Nephrol. 2014;15:29. doi:10.1186/1471-2369-15-29 24502751 PMC3927224

[16] Buysse DJ Thompson W Scott J, . Daytime symptoms in primary insomnia: a prospective analysis using ecological momentary assessment. Sleep Med. 2007;8(3):198–208. doi:10.1016/j.sleep.2006.10.006 17368098 PMC1899354

[17] Riis J Loewenstein G Baron J Jepson C Fagerlin A Ubel PA. Ignorance of hedonic adaptation to hemodialysis: a study using ecological momentary assessment. J Exp Psychol Gen. 2005;134(1):3–9. doi:10.1037/0096-3445.134.1.3 15702959

[18] Jhamb M Steel JL Yabes JG, . Effects of technology assisted stepped collaborative care intervention to improve symptoms in patients undergoing hemodialysis: the TĀCcare randomized clinical trial. JAMA Intern Med. 2023;183(8):795–805. doi:10.1001/jamainternmed.2023.2215 37338898 PMC10282960

[19] Roumelioti ME Steel JL Yabes J, . Rationale and design of technology assisted stepped collaborative care intervention to improve patient-centered outcomes in hemodialysis patients (TĀCcare trial). Contemp Clin Trials. 2018;73:81–91. doi:10.1016/j.cct.2018.09.002 30208343 PMC6168366

[20] Webster K Cella D Yost K. The functional assessment of chronic illness therapy (FACIT) measurement system: properties, applications, and interpretation. Health Qual Life Outcomes. 2003;1:79. doi:10.1186/1477-7525-1-79 14678568 PMC317391

[21] Wang YP Gorenstein C. Psychometric properties of the Beck depression inventory-II: a comprehensive review. Braz J Psychiatry. 2013;35(4):416–431. doi:10.1590/1516-4446-2012-1048 24402217

[22] Williams N. The GAD-7 questionnaire. Occup Med. 2014;64(3):224. doi:10.1093/occmed/kqt161

[23] Buysse DJ Reynolds CF Monk TH Berman SR Kupfer DJ. The Pittsburgh sleep quality index: a new instrument for psychiatric practice and research. Psychiatry Res. 1989;28(2):193–213. doi:10.1016/0165-1781(89)90047-4 2748771

[24] Charlson ME Carrozzino D Guidi J Patierno C. Charlson comorbidity index: a critical review of clinimetric properties. Psychother Psychosom. 2022;91(1):8–35. doi:10.1159/000521288 34991091

[25] Almutary H Douglas C Bonner A. Towards a symptom cluster model in chronic kidney disease: a structural equation approach. J Adv Nurs. 2017;73(10):2450–2461. doi:10.1111/jan.13303 28329420

[26] Jhamb M Abdel-Kader K Yabes J, . Comparison of fatigue, pain, and depression in patients with advanced kidney disease and cancer—symptom burden and clusters. J Pain Symptom Manage. 2019;57(3):566–575.e3. doi:10.1016/j.jpainsymman.2018.12.006 30552961 PMC6382584

[27] Ho SY Rohan KJ Parent J Tager FA McKinley PS. A longitudinal study of depression, fatigue, and sleep disturbances as a symptom cluster in women with breast cancer. J Pain Symptom Manage. 2015;49(4):707–715. doi:10.1016/j.jpainsymman.2014.09.009 25461671 PMC4380836

[28] Laird BJA Scott AC Colvin LA, . Pain, depression, and fatigue as a symptom cluster in advanced cancer. J Pain Symptom Manage. 2011;42(1):1–11. doi:10.1016/j.jpainsymman.2010.10.261 21402467

[29] Alvarez L Brown D Hu D Chertow GM Vassalotti JA Prichard S. Intradialytic symptoms and recovery time in patients on thrice-weekly in-center hemodialysis: a cross-sectional online survey. Kidney Med. 2020;2(2):125–130. doi:10.1016/j.xkme.2019.10.010 32734233 PMC7380355

[30] National Institute of Diabetes and Digestive and Kidney Diseases. A Scientific Workshop on Post-Dialysis Fatigue-2023; 2023. Accessed January 5, 2024. https://www.niddk.nih.gov/news/meetings-workshops/2023/scientific-workshop-on-post-dialysis-fatigue

[31] Chapman EJ Martino ED Edwards Z Black K Maddocks M Bennett MI. Practice review: evidence-based and effective management of fatigue in patients with advanced cancer. Palliat Med. 2022;36(1):7–14. doi:10.1177/02692163211046754 34903113 PMC8793304

[32] Jhamb M Weisbord SD Steel JL Unruh M. Fatigue in patients receiving maintenance dialysis: a review of definitions, measures, and contributing factors. Am J Kidney Dis. 2008;52(2):353–365. doi:10.1053/j.ajkd.2008.05.005 18572290 PMC2582327

[33] Davison SN Koncicki H Brennan F. Pain in chronic kidney disease: a scoping review. Semin Dial. 2014;27(2):188–204. doi:10.1111/sdi.12196 24517512

[34] Weisbord SD. Patient-Centered dialysis care: depression, pain, and quality of life. Semin Dial. 2016;29(2):158–164. doi:10.1111/sdi.12464 26748494

[35] Devaraj SM Roumelioti ME Yabes JG, . Correlates of rates and treatment readiness for depressive symptoms, pain, and fatigue in hemodialysis patients: results from the TĀCcare study. Kidney360. 2023;4(9):e1265–e1275. doi:10.34067/KID.0000000000000213 37461138 PMC10547226

