# Supplementary material for: Diurnal and Daily Symptom Variation in Patients with End Stage Kidney Disease: An Ecological Momentary Assessment Study
Source: Clin J Am Soc Nephrol. 2024 Jul 16;19(10):1292–300. doi: 10.2215/CJN.0000000000000524 (PMC11469768; doi:10.2215/CJN.0000000000000524)
Supplement: Supplementary file 1 [file cjasn-19-1292-s001.pdf]

ASN American Society of Nephrology | JASN Journal of the American Society of Nephrology | KIDNEY360 Accessing Our World From Every Angle | CJASN Clinical Journal of the American Society of Nephrology

ASN Journal Disclosure Form

As per ASN journal policy, I have disclosed any financial relationships or commitments I have held in the past 36 months as included below. I have listed my Current Employer below to indicate there is a relationship requiring disclosure. If no relationship exists, my Current Employer is not listed.

A. Alghwiri reports the following:
Employer: University of Pittsburgh

I understand that the information above will be published within the journal article, if accepted, and that failure to comply and/or to accurately and completely report the potential financial conflicts of interest could lead to the following: 1) Prior to publication, article rejection, or 2) Post-publication, sanctions ranging from, but not limited to, issuing a correction, reporting the inaccurate information to the authors' institution, banning authors from submitting work to ASN journals for varying lengths of time, and/or retraction of the published work.

Name: Alaa A. Alghwiri
Manuscript ID: CJASN-2024-000443R2
Manuscript Title: Diurnal and Daily Symptom Variation in Patients with End-Stage Kidney Disease: An Ecological Momentary Assessment Study,
Date of Completion: June 26, 2024
Disclosure Updated Date: May 16, 2024

ASN American Society of Nephrology | JASN Journal of the American Society of Nephrology | KIDNEY360 Accessing Our World From Every Angle | CJASN Clinical Journal of the American Society of Nephrology

ASN Journal Disclosure Form

As per ASN journal policy, I have disclosed any financial relationships or commitments I have held in the past 36 months as included below. I have listed my Current Employer below to indicate there is a relationship requiring disclosure. If no relationship exists, my Current Employer is not listed.

S. Erickson reports the following:
Employer: UNM

I understand that the information above will be published within the journal article, if accepted, and that failure to comply and/or to accurately and completely report the potential financial conflicts of interest could lead to the following: 1) Prior to publication, article rejection, or 2) Post-publication, sanctions ranging from, but not limited to, issuing a correction, reporting the inaccurate information to the authors' institution, banning authors from submitting work to ASN journals for varying lengths of time, and/or retraction of the published work.

Name: Sarah Jane Erickson
Manuscript ID: CJASN-2024-000443R2
Manuscript Title: Diurnal and Daily Symptom Variation in Patients with End-Stage Kidney Disease: An Ecological Momentary Assessment Study
Date of Completion: June 26, 2024
Disclosure Updated Date: May 15, 2024

ASN American Society of Nephrology
JASN Journal of the American Society of Nephrology
KIDNEY360 Accessing Our World From Every Angle
CJASN Clinical Journal of the American Society of Nephrology

ASN Journal Disclosure Form

As per ASN journal policy, I have disclosed any financial relationships or commitments I have held in the past 36 months as included below. I have listed my Current Employer below to indicate there is a relationship requiring disclosure. If no relationship exists, my Current Employer is not listed.

Z. Han reports the following:
Employer: University of Pittsburgh

I understand that the information above will be published within the journal article, if accepted, and that failure to comply and/or to accurately and completely report the potential financial conflicts of interest could lead to the following: 1) Prior to publication, article rejection, or 2) Post-publication, sanctions ranging from, but not limited to, issuing a correction, reporting the inaccurate information to the authors' institution, banning authors from submitting work to ASN journals for varying lengths of time, and/or retraction of the published work.

Name: Zhuoheng Han
Manuscript ID: CJASN-2024-000443R1
Manuscript Title: Diurnal and Daily Symptom Variation in Patients with End-Stage Kidney Disease: An Ecological Momentary Assessment Study
Date of Completion: June 20, 2024
Disclosure Updated Date: May 21, 2024

ASN American Society of Nephrology · JASN Journal of the American Society of Nephrology · KIDNEY360 Accessing Our World From Every Angle · CJASN Clinical Journal of the American Society of Nephrology

ASN Journal Disclosure Form

As per ASN journal policy, I have disclosed any financial relationships or commitments I have held in the past 36 months as included below. I have listed my Current Employer below to indicate there is a relationship requiring disclosure. If no relationship exists, my Current Employer is not listed.

M. Jhamb reports the following:
Employer: University of Pittsburgh and University of Pittsburgh Medical Center; Consultancy: Xcenda, LLC; Boehringer Ingelheim LLC, CKD Leaders Networks, Eli-Lilly; Research Funding: NIH, Dialysis Clinic, Inc., Bayer LLC, Pfizer, CKD Leaders Network; and Other Interests or Relationships: Member of ASN and National Kidney Foundation.

I understand that the information above will be published within the journal article, if accepted, and that failure to comply and/or to accurately and completely report the potential financial conflicts of interest could lead to the following: 1) Prior to publication, article rejection, or 2) Post-publication, sanctions ranging from, but not limited to, issuing a correction, reporting the inaccurate information to the authors' institution, banning authors from submitting work to ASN journals for varying lengths of time, and/or retraction of the published work.

Name: Manisha Jhamb
Manuscript ID: CJASN-2024-000443R2
Manuscript Title: Diurnal and Daily Symptom Variation in Patients with End-Stage Kidney Disease: An Ecological Momentary Assessment Study
Date of Completion: June 26, 2024
Disclosure Updated Date: April 29, 2024

ASN American Society of Nephrology | JASN Journal of the American Society of Nephrology | KIDNEY360 Accessing Our World From Every Angle | CJASN Clinical Journal of the American Society of Nephrology

ASN Journal Disclosure Form

As per ASN journal policy, I have disclosed any financial relationships or commitments I have held in the past 36 months as included below. I have listed my Current Employer below to indicate there is a relationship requiring disclosure. If no relationship exists, my Current Employer is not listed.

C. Kallem has nothing to disclose.

I understand that the information above will be published within the journal article, if accepted, and that failure to comply and/or to accurately and completely report the potential financial conflicts of interest could lead to the following: 1) Prior to publication, article rejection, or 2) Post-publication, sanctions ranging from, but not limited to, issuing a correction, reporting the inaccurate information to the authors' institution, banning authors from submitting work to ASN journals for varying lengths of time, and/or retraction of the published work.

Name: Cramer J Kallem
Manuscript ID: CJASN-2024-000443R1
Manuscript Title: Diurnal and Daily Symptom Variation in Patients with End-Stage Kidney Disease: An Ecological Momentary Assessment Study
Date of Completion: June 3, 2024
Disclosure Updated Date: April 23, 2024

ASN
American Society of Nephrology
JASN
Journal of the American Society of Nephrology
KIDNEY360
Accessing Our World From Every Angle
CJASN
Clinical Journal of the American Society of Nephrology

ASN Journal Disclosure Form

As per ASN journal policy, I have disclosed any financial relationships or commitments I have held in the past 36 months as included below. I have listed my Current Employer below to indicate there is a relationship requiring disclosure. If no relationship exists, my Current Employer is not listed.

M. Roumelioti reports the following:
Employer: University of New Mexico; Consultancy: My spouse: Quanta, Otsuka; Advisory or Leadership Role: Chair of the Medical Board ESRD Network 13; and Other Interests or Relationships: Participating in DCI quality meetings and receiving financial support.

I understand that the information above will be published within the journal article, if accepted, and that failure to comply and/or to accurately and completely report the potential financial conflicts of interest could lead to the following: 1) Prior to publication, article rejection, or 2) Post-publication, sanctions ranging from, but not limited to, issuing a correction, reporting the inaccurate information to the authors' institution, banning authors from submitting work to ASN journals for varying lengths of time, and/or retraction of the published work.

Name: Maria-Eleni Roumelioti
Manuscript ID: CJASN-2024-000443R1
Manuscript Title: Diurnal and Daily Symptom Variation in Patients with End-Stage Kidney Disease: An Ecological Momentary Assessment Study
Date of Completion: June 3, 2024
Disclosure Updated Date: May 8, 2024

ASN American Society of Nephrology
JASN Journal of the American Society of Nephrology
KIDNEY360 Accessing Our World From Every Angle
CJASN Clinical Journal of the American Society of Nephrology

ASN Journal Disclosure Form

As per ASN journal policy, I have disclosed any financial relationships or commitments I have held in the past 36 months as included below. I have listed my Current Employer below to indicate there is a relationship requiring disclosure. If no relationship exists, my Current Employer is not listed.

J. Steel reports the following:
Employer: University of Pittsburgh; and Patents or Royalties: Springer.

I understand that the information above will be published within the journal article, if accepted, and that failure to comply and/or to accurately and completely report the potential financial conflicts of interest could lead to the following: 1) Prior to publication, article rejection, or 2) Post-publication, sanctions ranging from, but not limited to, issuing a correction, reporting the inaccurate information to the authors' institution, banning authors from submitting work to ASN journals for varying lengths of time, and/or retraction of the published work.

Name: Jennifer L. Steel
Manuscript ID: CJASN-2024-000443R1
Manuscript Title: Diurnal and Daily Symptom Variation in Patients with End-Stage Kidney Disease: An Ecological Momentary Assessment Study
Date of Completion: July 2, 2024
Disclosure Updated Date: March 4, 2024

ASN American Society of Nephrology
JASN Journal of the American Society of Nephrology
KIDNEY360 Accessing Our World From Every Angle
CJASN Clinical Journal of the American Society of Nephrology

ASN Journal Disclosure Form

As per ASN journal policy, I have disclosed any financial relationships or commitments I have held in the past 36 months as included below. I have listed my Current Employer below to indicate there is a relationship requiring disclosure. If no relationship exists, my Current Employer is not listed.

M. Unruh reports the following:
Employer: University of New Mexico; New Mexico Veterans Hospital; and Research Funding: Dialysis Clinic Inc.

I understand that the information above will be published within the journal article, if accepted, and that failure to comply and/or to accurately and completely report the potential financial conflicts of interest could lead to the following: 1) Prior to publication, article rejection, or 2) Post-publication, sanctions ranging from, but not limited to, issuing a correction, reporting the inaccurate information to the authors' institution, banning authors from submitting work to ASN journals for varying lengths of time, and/or retraction of the published work.

Name: Mark L. Unruh
Manuscript ID: CJASN-2024-000443R1
Manuscript Title: Diurnal and Daily Symptom Variation in Patients with End-Stage Kidney Disease: An Ecological Momentary Assessment Study
Date of Completion: June 20, 2024
Disclosure Updated Date: June 20, 2024

ASN
American Society of Nephrology
JASN
Journal of the American Society of Nephrology
KIDNEY360
Accessing Our World From Every Angle
CJASN
Clinical Journal of the American Society of Nephrology

ASN Journal Disclosure Form

As per ASN journal policy, I have disclosed any financial relationships or commitments I have held in the past 36 months as included below. I have listed my Current Employer below to indicate there is a relationship requiring disclosure. If no relationship exists, my Current Employer is not listed.

J. Yabes reports the following:
Employer: University of Pittsburgh

I understand that the information above will be published within the journal article, if accepted, and that failure to comply and/or to accurately and completely report the potential financial conflicts of interest could lead to the following: 1) Prior to publication, article rejection, or 2) Post-publication, sanctions ranging from, but not limited to, issuing a correction, reporting the inaccurate information to the authors' institution, banning authors from submitting work to ASN journals for varying lengths of time, and/or retraction of the published work.

Name: Jonathan Guerrero Yabes
Manuscript ID: CJASN-2024-000443R1
Manuscript Title: Diurnal and Daily Symptom Variation in Patients with End-Stage Kidney Disease: An Ecological Momentary Assessment Study
Date of Completion: June 20, 2024
Disclosure Updated Date: May 8, 2024
