# Supplementary material for: Diurnal and Daily Symptom Variation in Patients with End Stage Kidney Disease: An Ecological Momentary Assessment Study
Source: Clin J Am Soc Nephrol. 2024 Jul 16;19(10):1292–300. doi: 10.2215/CJN.0000000000000524 (PMC11469768; doi:10.2215/CJN.0000000000000524)
Supplement: Supplementary file 2 [file cjasn-19-1292-s002.pdf]

Supplemental Online Content

Diurnal and Daily Symptom Variation in Patients with End-Stage Kidney Disease: An Ecological Momentary Assessment Study

Cramer J. Kallem, PsyD
Alaa A. Alghwiri, PhD
Jonathan G. Yabes, PhD
Sarah Erickson, PhD
Zhuoheng Han, MSP
Maria-Eleni Roumelioti, MD
Jennifer L. Steel, PhD
Manisha Jhamb, MD, MPH
Mark Unruh, MD, MS

Table of Contents:

Supplemental Table 1. Unadjusted Changes in Symptom Domain Scores between Time Points

Supplemental Table 2. Changes in Symptom Domain Scores between Time Points after adjusting for age, race, gender, and Charlson Comorbidity Index

Supplemental Table 3. Unadjusted Mean Difference in Symptom Domain Scores on Dialysis versus Non-Dialysis Days at all Time Points

Supplemental Table 4. Mean Difference in Symptom Domain Scores on Dialysis versus Non-Dialysis Days at all Time Points after adjusting for age, race, gender, and Charlson Comorbidity Index

Supplemental Figure 1a. Subgroup Analysis of DISS Alert Cognition Scores

Supplemental Figure 1b. Subgroup Analysis of DISS Sleepiness/Fatigue Scores

Supplemental Figure 1c. Subgroup Analysis of DISS Positive Mood Scores

Supplemental Figure 1d. Subgroup Analysis of DISS Negative Mood Scores

Supplemental Figure 2. Predicted Means of Symptom Domain Scores for Early Morning Hemodialysis Patients by Time of Day on Dialysis vs. Non-Dialysis Days after Controlling for Age, Race, Sex, and Comorbidity Burden

Supplemental Figure 3. Predicted Means of Symptom Domain Scores for Afternoon Hemodialysis Patients by Time of Day on Dialysis vs. Non-Dialysis Days after Controlling for Age, Race, Sex, and Comorbidity Burden

Supplemental Table 1: Unadjusted Changes in Symptom Domain Scores between Time Points

Symptom Domain	Time Comparison	Mean Difference	95% CI	p-value
Hemodialysis Days				
AC	Morning – Early Afternoon	-0.11	-0.21, -0.01	0.04*
	Early Afternoon – Late Afternoon	0.08	-0.02, 0.17	0.11
	Late Afternoon – Evening	0.02	-0.08, 0.11	0.74
SF	Morning – Early Afternoon	0.38	0.29, 0.57	<0.001**
	Early Afternoon – Late Afternoon	-0.07	0.25, 0.11	0.43
	Late Afternoon – Evening	0.22	0.04, 0.39	0.02*
PM	Morning – Early Afternoon	-0.12	-0.26, 0.01	0.08
	Early Afternoon – Late Afternoon	0.06	-0.08, 0.19	0.41
	Late Afternoon – Evening	0.09	-0.04, 0.22	0.18
NM	Morning – Early Afternoon	0.17	0.03, 0.30	0.02*
	Early Afternoon – Late Afternoon	-0.02	-0.15, 0.11	0.75
	Late Afternoon – Evening	-0.13	0.26, -0.01	0.04*
Non-Hemodialysis Days				
AC	Morning – Early Afternoon	-0.06	-0.15, 0.02	0.15
	Early Afternoon – Late Afternoon	-0.02	-0.10, 0.06	0.56
	Late Afternoon – Evening	0.05	-0.03, 0.13	0.20
SF	Morning – Early Afternoon	0.06	-0.09, 0.22	0.43
	Early Afternoon – Late Afternoon	0.14	0.01, 0.29	0.07
	Late Afternoon – Evening	0.35	0.20, 0.49	<0.001**
PM	Morning – Early Afternoon	-0.05	-0.16, 0.07	0.44
	Early Afternoon – Late Afternoon	-0.00	-0.11, 0.11	0.95
	Late Afternoon – Evening	-0.02	-0.13, 0.09	0.71
NM	Morning – Early Afternoon	0.02	-0.10, 0.13	0.77
	Early Afternoon – Late Afternoon	0.03	-0.08, 0.14	0.61
	Late Afternoon – Evening	0.08	-0.03, 0.19	0.16

Abbreviations: Alert Cognition (AC), Sleepiness/Fatigue (SF), Positive Mood (PM), Negative Mood (NM).

Note: Positive mean differnce = increase in symptom domain score from timepoint to timepoint; negative mean differnce = decrease in symptom domain score from timepoint to timepoint; *=p<0.05; **=p<0.001.

Supplemental Table 2: Changes in Symptom Domain Scores between Time Points after adjusting for age, race, gender, and Charlson Comorbidity Index

Symptom Domain	Time Comparison	Mean Difference	95% CI	p-value
Hemodialysis Days				
AC	Morning – Early Afternoon	-0.09	-0.20, 0.01	0.08
	Early Afternoon – Late Afternoon	0.06	-0.03, 0.16	0.19
	Late Afternoon – Evening	0.02	-0.07, 0.11	0.65
SF	Morning – Early Afternoon	0.41	0.23, 0.60	<0.001**
	Early Afternoon – Late Afternoon	-0.07	0.25, 0.11	0.45
	Late Afternoon – Evening	0.22	0.04, 0.39	0.02*
PM	Morning – Early Afternoon	-0.12	-0.25, 0.02	0.10
	Early Afternoon – Late Afternoon	0.04	-0.10, 0.17	0.60
	Late Afternoon – Evening	0.10	-0.03, 0.23	0.14
NM	Morning – Early Afternoon	0.17	0.03, 0.30	0.02*
	Early Afternoon – Late Afternoon	-0.01	-0.15, 0.12	0.86
	Late Afternoon – Evening	-0.14	-0.26, -0.01	0.04*
Non-Hemodialysis Days				
AC	Morning – Early Afternoon	-0.07	-0.15, 0.02	0.15
	Early Afternoon – Late Afternoon	-0.02	-0.10, 0.06	0.55
	Late Afternoon – Evening	0.05	-0.03, 0.12	0.24
SF	Morning – Early Afternoon	0.08	-0.08, 0.24	0.33
	Early Afternoon – Late Afternoon	0.14	-0.01, 0.29	0.07
	Late Afternoon – Evening	0.35	0.20, 0.50	<0.001**
PM	Morning – Early Afternoon	-0.03	-0.14, 0.09	0.61
	Early Afternoon – Late Afternoon	-0.01	-0.12, 0.10	0.84
	Late Afternoon – Evening	-0.02	-0.13, 0.09	0.68
NM	Morning – Early Afternoon	0.01	-0.11, 0.12	0.91
	Early Afternoon – Late Afternoon	0.02	-0.09, 0.14	0.67
	Late Afternoon – Evening	0.07	-0.04, 0.18	0.19

Abbreviations: Alert Cognition (AC), Sleepiness/Fatigue (SF), Positive Mood (PM), Negative Mood (NM).

Note: Positive mean differnce = increase in symptom domain score from timepoint to timepoint; negative mean differnce = decrease in symptom domain score from timepoint to timepoint; *=p<0.05; **=p<0.001.

Supplemental Table 3: Unadjusted Mean Difference in Symptom Domain Scores on Dialysis versus Non-Dialysis Days at all Time Points

Symptom Domain	Time Point	Mean Difference	95% CI	p-value
**AC**	Morning	0.13	0.03, 0.22	0.01*
	Early Afternoon	0.17	0.09, 0.26	<0.001**
	Late Afternoon	0.07	-0.01, 0.16	0.10
	Evening	0.11	0.02, 0.19	0.01*
**SF**	Morning	-0.33	-0.50, -0.17	<0.001**
	Early Afternoon	-0.65	-0.81, -0.49	<0.001**
	Late Afternoon	-0.44	-0.60, -0.28	<0.001**
	Evening	-0.31	-0.46, -0.16	<0.001**
**PM**	Morning	0.20	0.07, 0.33	0.002*
	Early Afternoon	0.28	0.16, 0.40	<0.001**
	Late Afternoon	0.22	0.10, 0.34	<0.001**
	Evening	0.11	-0.01, 0.23	0.07
**NM**	Morning	-0.11	-0.23, 0.02	0.10
	Early Afternoon	-0.26	-0.38, -0.14	<0.001**
	Late Afternoon	-0.21	-0.32, -0.09	0.001*
	Evening	0.00	-0.11, 0.12	0. 96

Abbreviations: Alert Cognition (AC), Sleepiness/Fatigue (SF), Positive Mood (PM), Negative Mood (NM).

Note: Positive mean difference = higher symptom domain score on dialysis days compared to non-dialysis days; negative mean difference = lower symptom domain score on dialysis days compared to non-dialysis days; *=p<0.05; **=p<0.001.

Supplemental Table 4: Mean Difference in Symptom Domain Scores on Dialysis versus Non-Dialysis Days at all Time Points after adjusting for age, race, gender, and Comorbidity Burden

Symptom Domain	Time Point	Mean Difference	95% CI	p-value
**AC**	Morning	0.13	0.04, 0.22	0.01*
	Early Afternoon	0.17	0.09, 0.26	<0.001**
	Late Afternoon	0.07	-0.01, 0.16	0.10
	Evening	0.11	0.02, 0.19	0.01*
**SF**	Morning	-0.33	-0.50, -0.16	<0.001**
	Early Afternoon	-0.65	-0.81, -0.49	<0.001**
	Late Afternoon	-0.44	-0.60, -0.28	<0.001**
	Evening	-0.31	-0.47, -0.16	<0.001**
**PM**	Morning	0.20	0.07, 0.33	0.002*
	Early Afternoon	0.28	0.16, 0.40	<0.001**
	Late Afternoon	0.22	0.10, 0.34	<0.001**
	Evening	0.11	-0.01, 0.23	0.07
**NM**	Morning	-0.10	-0.23, 0.02	0.10
	Early Afternoon	-0.26	-0.38, -0.13	<0.001**
	Late Afternoon	-0.21	-0.32, -0.09	0.001*
	Evening	0.00	-0.11, 0.12	0. 96

Abbreviations: Alert Cognition (AC), Sleepiness/Fatigue (SF), Positive Mood (PM), Negative Mood (NM).

Note: Positive mean difference = higher symptom domain score on dialysis days compared to non-dialysis days; negative mean difference = lower symptom domain score on dialysis days compared to non-dialysis days; *=p<0.05; **=p<0.001.

Supplemental Figure 1a: Subgroup Analysis of DISS Alert Cognition Scores

Subgroup	n	Mean (95% CI)
Age		
< 65	112	4.98 (4.84, 5.12)
>= 65	48	4.94 (4.74, 5.14)
Gender		
Female	72	5.07 (4.90, 5.24)
Male	88	4.88 (4.73, 5.04)
Race		
Black	45	5.01 (4.84, 5.17)
Others	32	5.15 (4.81, 5.49)
White	83	4.87 (4.72, 5.03)
Ethnicity		
Hispanic	28	4.89 (4.56, 5.22)
Non-Hispanic	132	4.98 (4.86, 5.10)
CCI		
< 4	33	4.71 (4.47, 4.94)
>= 4	127	5.04 (4.91, 5.16)
Years on Dialysis		
< 1	36	4.88 (4.64, 5.12)
>= 1	124	4.99 (4.86, 5.12)
Dialysis Shift		
Midday	80	4.96 (4.80, 5.12)
Morning	75	4.99 (4.82, 5.15)
Dialysis Schedule		
M/W/F	103	4.98 (4.84, 5.13)
T/Th/S	57	4.94 (4.76, 5.13)

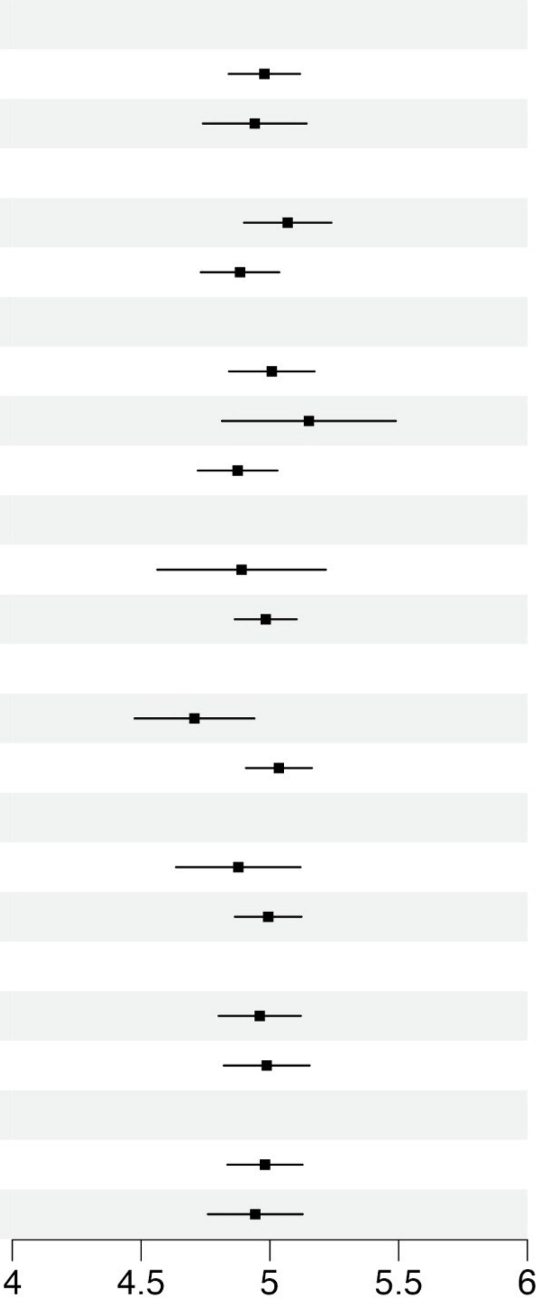

Abbreviations: Daytime Insomnia Symptom Scale (DISS), Charlson Comorbidity Index (CCI), Monday/Wednesday/Friday (M/W/F), Tuesday/Thursday/Saturday (T/Th/S).

Note: Lower Alert Cognition scores are worse. There were no significant differences in Alert Cognition scores between subgroups. Patients on an evening dialysis shift were excluded from the dialysis shift analysis due to small sample size (N=5).

Supplemental Figure 1b: Subgroup Analysis of DISS Sleepiness/Fatigue Scores

Subgroup	n	Mean (95% CI)
Age		
< 65	112	3.92 (3.70, 4.14)
>= 65	48	3.66 (3.33, 3.98)
Gender		
Female	72	3.77 (3.49, 4.06)
Male	88	3.90 (3.65, 4.14)
Race		
Black	45	3.68 (3.32, 4.05)
Others	32	3.63 (3.20, 4.06)
White	83	4.01 (3.76, 4.26)
Ethnicity		
Hispanic	28	4.09 (3.66, 4.53)
Non-Hispanic	132	3.79 (3.59, 3.99)
CCI		
< 4	33	4.33 (3.97, 4.70)
>= 4	127	3.71 (3.51, 3.92)
Years on Dialysis		
< 1	36	3.67 (3.28, 4.06)
>= 1	124	3.89 (3.68, 4.10)
Dialysis Shift		
Midday	80	3.88 (3.62, 4.14)
Morning	75	3.72 (3.46, 3.98)
Dialysis Schedule		
M/W/F	103	3.77 (3.54, 4.00)
T/Th/S	57	3.97 (3.67, 4.27)

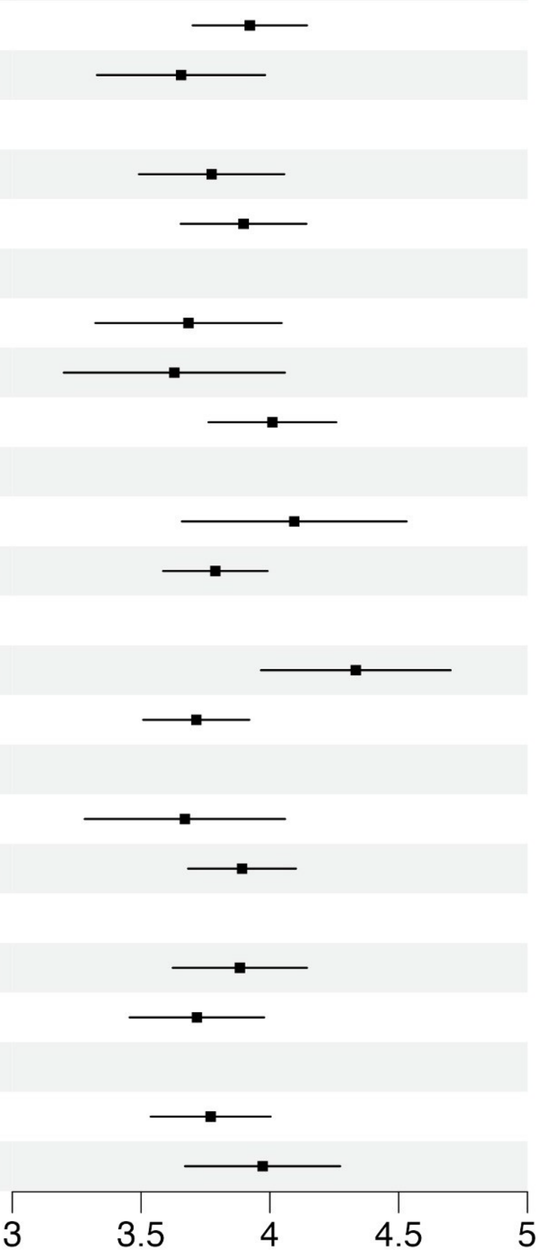

Abbreviations: Daytime Insomnia Symptom Scale (DISS), Charlson Comorbidity Index (CCI), Monday/Wednesday/Friday (M/W/F), Tuesday/Thursday/Saturday (T/Th/S).

Note: Higher Sleepiness/Fatigue scores are worse. There were no significant differences in Sleepiness/Fatigue scores between subgroups. Patients on an evening dialysis shift were excluded from the dialysis shift analysis due to small sample size (N=5).

Supplemental Figure 1c: Subgroup Analysis of DISS Positive Mood Scores

Subgroup	n	Mean (95% CI)
Age		
< 65	112	4.43 (4.23, 4.62)
>= 65	48	4.44 (4.19, 4.70)
Gender		
Female	72	4.41 (4.18, 4.64)
Male	88	4.45 (4.24, 4.66)
Race*		
Black	45	4.36 (4.08, 4.63)
Others	32	4.89 (4.48, 5.30)
White	83	4.30 (4.10, 4.50)
Ethnicity		
Hispanic	28	4.34 (3.91, 4.76)
Non-Hispanic	132	4.45 (4.29, 4.62)
CCI		
< 4	33	4.13 (3.80, 4.47)
>= 4	127	4.51 (4.34, 4.68)
Years on Dialysis		
< 1	36	4.48 (4.15, 4.81)
>= 1	124	4.42 (4.24, 4.59)
Dialysis Shift		
Midday	80	4.30 (4.08, 4.51)
Morning	75	4.58 (4.35, 4.81)
Dialysis Schedule		
M/W/F	103	4.48 (4.29, 4.68)
T/Th/S	57	4.34 (4.07, 4.60)

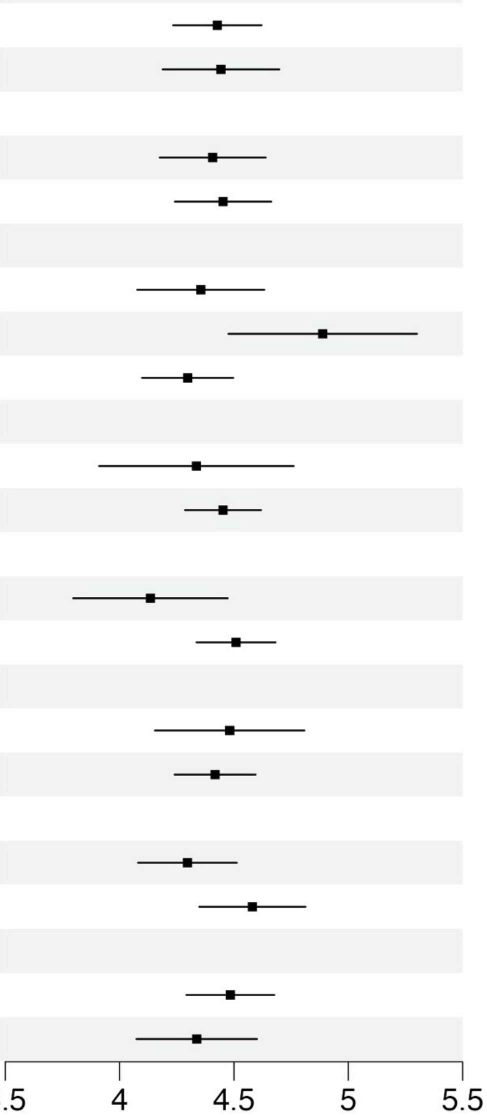

Abbreviations: Daytime Insomnia Symptom Scale (DISS), Charlson Comorbidity Index (CCI), Monday/Wednesday/Friday (M/W/F), Tuesday/Thursday/Saturday (T/Th/S).

Note: Lower Positive Mood Scores are worse. *There were significant differences in Positive Mood scores between race subgroups with patients in the other race category (American Indian, >1 race, or unknown) having higher scores (mean= 4.89 ± 1.14) than Black (mean= 4.36 ± 0.93) or White patients (mean= 4.30 ± 0.91, p=0.03). There were no other significant differences in Positive Mood scores between subgroups. Patients on an evening dialysis shift were excluded from the dialysis shift analysis due to small sample size (N=5).

Supplemental Figure 1d: Subgroup Analysis of DISS Negative Mood Scores

Subgroup	n	Mean (95% CI)
Age*		
< 65	112	2.64 (2.43, 2.86)
>= 65	48	2.21 (1.93, 2.49)
Gender		
Female	72	2.43 (2.19, 2.67)
Male	88	2.58 (2.33, 2.84)
Race		
Black	45	2.43 (2.12, 2.75)
Others	32	2.36 (1.95, 2.77)
White	83	2.62 (2.36, 2.87)
Ethnicity		
Hispanic	28	2.62 (2.21, 3.02)
Non-Hispanic	132	2.49 (2.30, 2.69)
CCI		
< 4	33	2.86 (2.48, 3.24)
>= 4	127	2.42 (2.23, 2.62)
Years on Dialysis		
< 1	36	2.37 (2.01, 2.73)
>= 1	124	2.56 (2.35, 2.76)
Dialysis Shift		
Midday	80	2.62 (2.37, 2.88)
Morning	75	2.36 (2.11, 2.61)
Dialysis Schedule		
M/W/F	103	2.50 (2.28, 2.72)
T/Th/S	57	2.54 (2.24, 2.85)

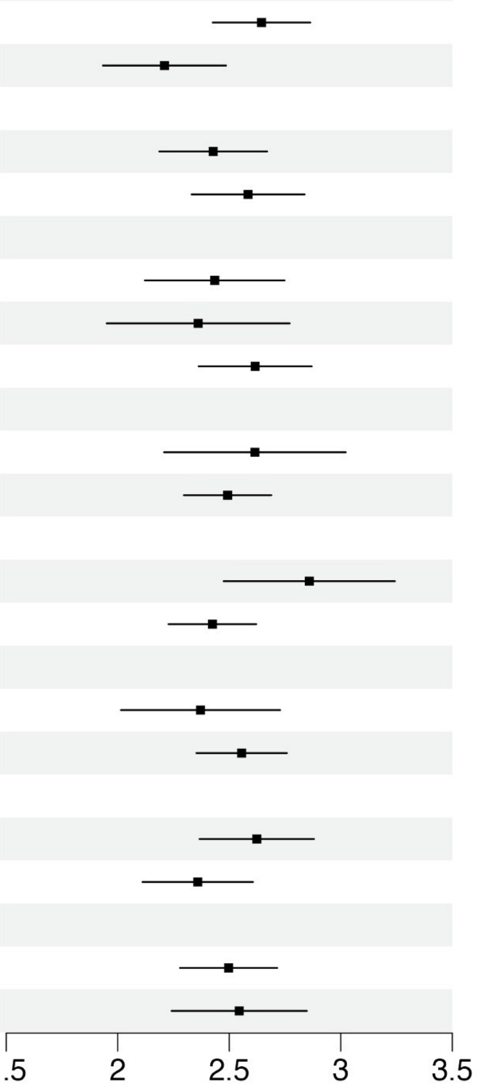

Abbreviations: Daytime Insomnia Symptom Scale (DISS), Charlson Comorbidity Index (CCI), Monday/Wednesday/Friday (M/W/F), Tuesday/Thursday/Saturday (T/Th/S).

Note: Higher Negative Mood scores are worse. *There were significant differences in Negative Mood scores between age subgroups with patients under the age of 65 having higher scores (mean= 2.64 ± 1.17) than those age 65 or older (mean= 2.21 ± 0.95, p=0.04). There were no other significant differences in Negative Mood scores between subgroups. Patients on an evening dialysis shift were excluded from the dialysis shift analysis due to small sample size (N=5).

Supplemental Figure 2. Predicted Means of Symptom Domain Scores for Early Morning Hemodialysis Patients by Time of Day on Dialysis vs. Non-Dialysis Days after Controlling for Age, Race, Sex, and Comorbidity Burden

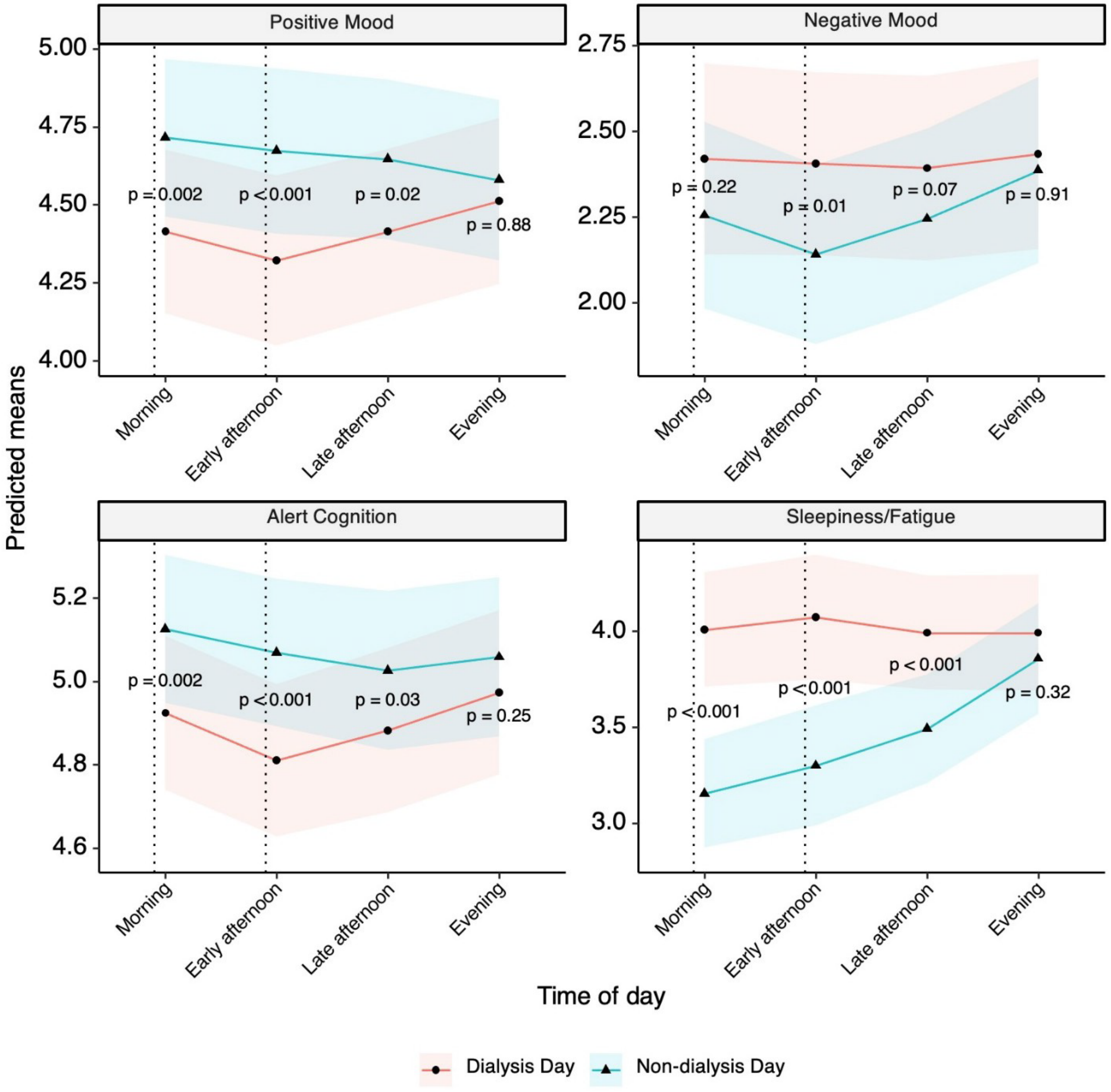

Note: P-values are based on adjusted mixed model comparisons of mean symptom domain scores at each time point on dialysis versus non-dialysis days. Dotted vertical lines indicate the average start and end times for early morning hemodialysis treatments (mean dialysis start time = 6:14 AM ± 30 minutes, mean dialysis end time = 10:18 AM ± 42 minutes). For Alert Cognition and Positive mood, higher score is better; for Negative Mood and Sleepiness/Fatigue, higher score is worse.

Supplemental Figure 3. Predicted Means of Symptom Domain Scores for Afternoon Hemodialysis Patients by Time of Day on Dialysis vs. Non-Dialysis Days after Controlling for Age, Race, Sex, and Comorbidity Burden

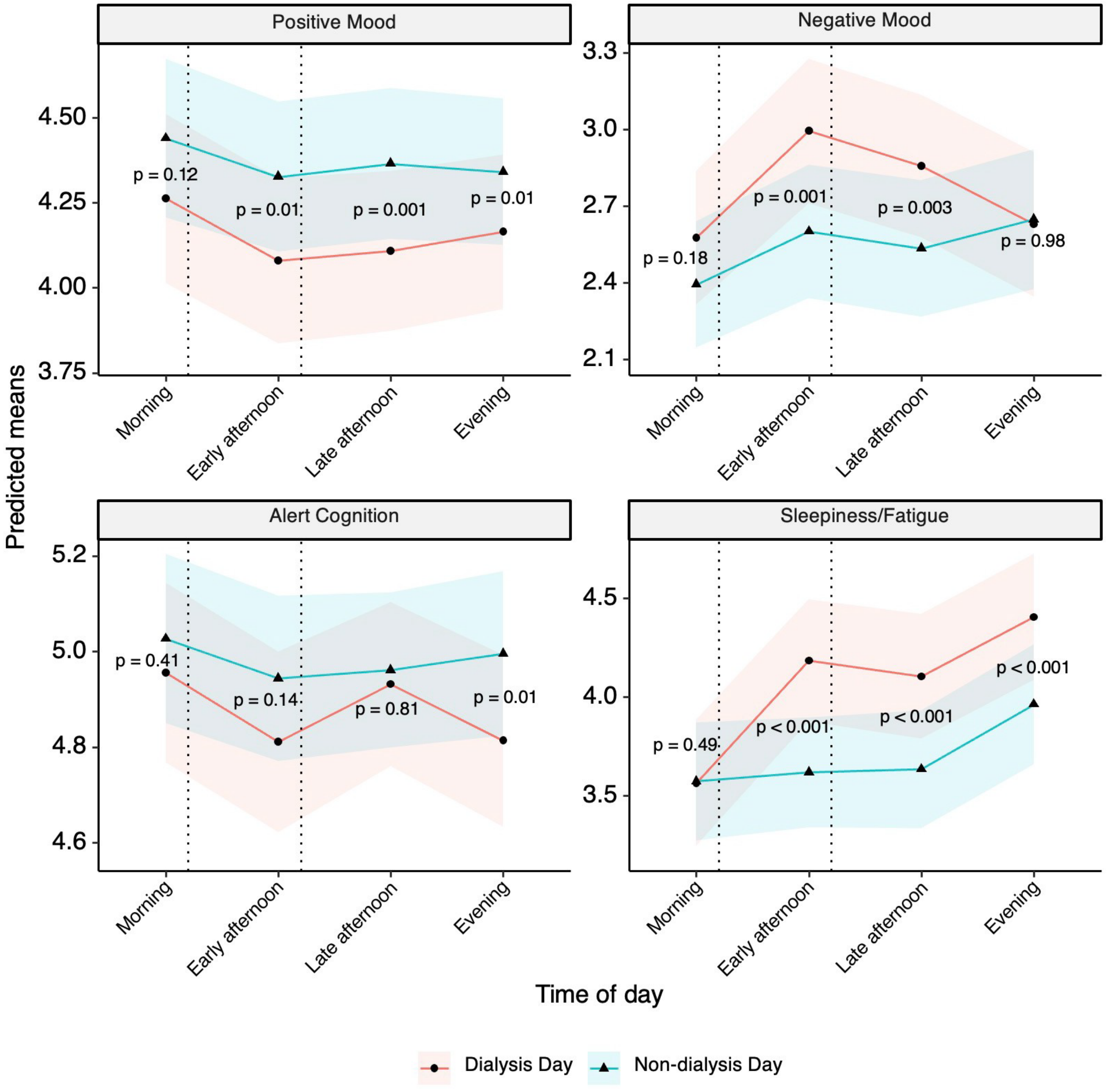

Note: P-values are based on adjusted mixed model comparisons of mean symptom domain scores at each time point on dialysis versus non-dialysis days. Dotted vertical lines indicate the average start and end times for afternoon hemodialysis treatments (mean dialysis start time = 10:57 AM ± 46 minutes, mean dialysis end time = 3:02 PM ± 50 minutes). For Alert Cognition and Positive mood, higher score is better; for Negative Mood and Sleepiness/Fatigue, higher score is worse.
